# Clinical Breakthrough in Targeted Protein Degradation: Reflections on the Approval of ARV‐471

**DOI:** 10.1002/cai2.70068

**Published:** 2026-06-07

**Authors:** Yu Rao

**Affiliations:** ^1^ MOE Key Laboratory of Protein Sciences, School of Pharmaceutical Sciences, MOE Key Laboratory of Bioorganic Phosphorus Chemistry and Chemical Biology, State Key Laboratory of Molecular Oncology Tsinghua University Beijing China; ^2^ Beijing Key Laboratory of Targeted Protein Degradation and Molecular Glue Therapeutics Tsinghua University Beijing China

**Keywords:** breast cancer, protac, targeted protein degradation, vepdegestrant

On May 1, 2026, the US Food and Drug Administration approved vepdegestrant (ARV‐471; Veppanu), developed by Arvinas in partnership with Pfizer, for the treatment of ESR1‐mutated, ER‐positive/HER2‐negative advanced or metastatic breast cancer [1]. As the first PROTAC (proteolysis‐targeting chimera) drug to reach the market, ARV‐471 represents the culmination of more than two decades of scientific development, translating the concept of targeted protein degradation from academic laboratories into a clinically viable therapeutic modality. Its approval establishes a critical regulatory and commercial precedent for the broader class of degraders now advancing through pharmaceutical pipelines (Figure 1).

**Figure 1:**
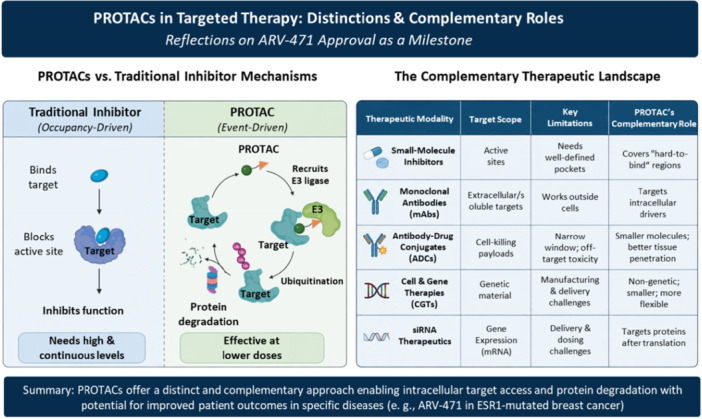
Mechanistic distinction and complementary therapeutic role of PROTACs. Created in BioRender. CHENG, L. (2026) https://BioRender.com/zgq3amd.

Traditional inhibitors act through occupancy‐driven target blockade, whereas PROTACs function through an event‐driven mechanism by recruiting an E3 ligase to induce ubiquitination and proteasomal degradation of the target protein. Compared with antibodies, antibody‐drug conjugates (ADCs), cell and gene therapies (CGTs), and siRNA therapeutics, PROTACs provide a complementary small‐molecule strategy for accessing intracellular proteins and eliminating disease‐associated targets after protein synthesis.

The mechanism of PROTACs differs fundamentally from that of conventional small‐molecule inhibitors. Traditional inhibitors operate through an occupancy‐driven model, in which the drug molecule must bind to and block the active site of a target protein, typically requiring sustained high local concentrations to maintain efficacy. This approach struggles against targets lacking well‐defined catalytic pockets—such as transcription factors, scaffold proteins, and many non‐enzymatic signaling molecules—that have long been considered “undruggable.” PROTACs, by contrast, function through an event‐driven mechanism. These heterobifunctional molecules bind the target protein at one end and recruit an E3 ubiquitin ligase at the other, inducing the formation of a ternary complex that hijacks the cell's endogenous ubiquitin‐proteasome system to mark the disease‐associated protein for degradation. Because a single PROTAC molecule can catalytically mediate the destruction of multiple target molecules, effective doses are generally lower than those required for traditional inhibitors. Moreover, PROTACs do not require high‐affinity engagement of an active site; even modest binding to accessible surface regions can trigger degradation, substantially expanding the range of the proteome that can be pharmacologically addressed [2].

The approval of ARV‐471 was supported by data from the Phase III VERITAC‐2 trial. In patients with ESR1‐mutated breast cancer who had progressed following prior treatment with CDK4/6 inhibitors and endocrine therapy, vepdegestrant monotherapy achieved a median progression‐free survival (PFS) of 5.0 months, compared with 2.1 months for fulvestrant, representing a 43% reduction in the risk of disease progression or death [3]. ESR1 mutations represent a key resistance mechanism in ER‐positive breast cancer, enabling ligand‐independent receptor activation [4]. While the selective estrogen receptor degrader (SERD) fulvestrant can partially downregulate ER expression, the receptor degradation is incomplete and its monthly intramuscular administration poses adherence challenges. ARV‐471 achieves more profound ER degradation via oral administration, blocking oncogenic signaling closer to its source while improving patient convenience. These results validate the efficacy and safety of PROTAC‐based therapy in solid tumors and demonstrate that such molecules can be manufactured at scale with acceptable quality control standards.

Within the increasingly diverse landscape of modern therapeutics, PROTACs occupy a complementary position relative to monoclonal antibodies, ADCs, CGTs, and siRNA‐based medicines. Antibodies primarily target cell‐surface antigens or soluble ligands and are ineffective against intracellular proteins. ADCs combine antibody‐mediated targeting with cytotoxic payloads, yet their therapeutic window remains constrained by linker stability, payload off‐target toxicity, and heterogeneous solid‐tumor penetration. CGTs offer potentially curative interventions through genetic modification but involve complex manufacturing, high costs, and formidable challenges in solid‐tumor delivery and long‐term safety monitoring. siRNA therapeutics silence gene expression through RNA interference; while GalNAc‐conjugated liver delivery has matured, efficient targeting of extrahepatic tissues and the feasibility of chronic dosing regimens remain unresolved.

PROTACs, as chemically synthesized small molecules, offer oral bioavailability, strong tissue penetration, and relatively low production costs. Their ability to selectively eliminate intracellular proteins fills a gap that biologics cannot address. In oncology, ADCs may eradicate cell populations expressing high levels of surface antigens, while PROTACs simultaneously dismantle intracellular survival pathways. From a pharmacoeconomic perspective, PROTACs avoid the cold‐chain logistics and complex infusion infrastructure required for CGTs and ADCs. Rather than competing directly with these modalities, PROTACs are best understood as expanding the pharmacological toolkit, enabling rational combination strategies against complex diseases.

Looking forward, the application of PROTAC technology is extending well beyond oncology into autoimmune and neurodegenerative diseases. In autoimmunity, degraders targeting IRAK4 and BTK have shown particular promise. IRAK4 serves as a critical node in TLR/IL‐1R signaling and possesses both kinase activity and essential scaffolding functions. Conventional kinase inhibitors can only block catalytic activity, whereas PROTAC‐mediated degradation eliminates the entire protein, thereby interrupting scaffold‐dependent signal transduction and achieving more comprehensive suppression of inflammatory pathways. IRAK4‐targeting PROTACs, including Kymera Therapeutics’ KT‐474 and Simcere Pharmaceutical's SIM0711, have entered clinical trials or investigational new drug applications for indications such as hidradenitis suppurativa and atopic dermatitis [5]. BTK‐targeting degraders have also demonstrated superior efficacy compared with inhibition alone in preclinical models of systemic lupus erythematosus.

In neurodegeneration, Arvinas has developed ARV‐102, a brain‐penetrant PROTAC designed to degrade leucine‐rich repeat kinase 2 (LRRK2), a protein strongly implicated in Parkinson's disease. Early clinical data indicate that ARV‐102 achieves greater than 50% LRRK2 degradation in patients' cerebrospinal fluid and reduces associated biomarkers of neuroinflammation [6]. Meanwhile, PROTAC candidates directed against historically undruggable oncogenic targets such as KRAS G12D and BCL6 have entered early‐phase clinical testing.

The emergence of degrader‐antibody conjugates (DACs) represents another important frontier. By conjugating a PROTAC payload to a tumor‐targeting antibody, DACs aim to exploit the tissue specificity of antibody delivery to concentrate degraders within the tumor microenvironment while minimizing systemic exposure. The collaboration between C4 Therapeutics and Roche, announced in early 2026, signals that DACs are transitioning from conceptual exploration toward industrial development. Alongside PROTACs, molecular glues—monofunctional degraders that reshape E3 ligase substrate specificity—continue to advance, with the two modalities together defining the broader field of targeted protein degradation.

From the first report of peptide‐based PROTACs in 2001 to the approval of ARV‐471 in 2026, the journey has spanned a quarter‐century [7]. The clinical validation provided by ARV‐471 extends far beyond the treatment of breast cancer; it confirms that pharmacological protein degradation is a robust, manufacturable, and regulatorily approvable strategy. As additional candidates advance through late‐stage development and novel delivery formats such as DACs mature, targeted protein degradation is poised to play an increasingly important role across oncology, autoimmune disorders, and neurodegenerative diseases.

## Author Contributions

**Yu Rao:** writing – original draft (lead), writing – review and editing (lead), supervision (lead).

## Ethics Statement

The author has nothing to report.

## Consent

The author has nothing to report.

## Conflicts of Interest

Yu Rao is a member of the *Cancer Innovation* Editorial Board. To minimize bias, he was excluded from all editorial decision‐making related to the acceptance of this article for publication.

## Data Availability

Data sharing is not applicable to this article as no data sets were generated or analyzed during the current study.
